# Progressive Fibrotic Pneumonitis Following Durvalumab Therapy: A Challenging Case

**DOI:** 10.7759/cureus.100981

**Published:** 2026-01-07

**Authors:** Susana Viana, Isabel Monteiro, Marta Vilaça, Inês Neves, Raquel Calisto

**Affiliations:** 1 Internal Medicine, Hospital Pedro Hispano, Matosinhos, PRT; 2 Oncology, Hospital Pedro Hispano, Matosinhos, PRT; 3 Pulmonology, Hospital Pedro Hispano, Matosinhos, PRT

**Keywords:** durvalumab, immune checkpoint inhibitors, immune-related pneumonitis, nintedanib, non-small cell lung cancer

## Abstract

Durvalumab following chemoradiotherapy (CRT) is the standard of care for unresectable stage III non-small cell lung cancer (NSCLC). Immune-related pneumonitis (IrP) is a clinically relevant immune-related adverse event (irAE) that may limit treatment benefit and, although usually steroid-responsive, can rarely progress to chronic fibrotic lung disease.

We report the case of a 71-year-old man with stage IIIc squamous NSCLC treated with CRT followed by consolidation durvalumab. After the second durvalumab cycle, he developed worsening dyspnoea, and computed tomography (CT) showed bilateral multifocal ground-glass opacities, consistent with IrP, without tumour progression. Durvalumab was discontinued, and high-dose corticosteroids were initiated. Despite initial partial improvement, the patient deteriorated during steroid taper, developing hypoxemic respiratory failure. A new CT demonstrated progressive interstitial involvement with evolution towards fibrotic changes. Escalation to intravenous methylprednisolone pulses led to improved gas exchange, although dyspnoea persisted. Nintedanib was introduced as an antifibrotic therapy, achieving radiographic stabilisation. Nonetheless, clinical improvement remained limited, and four months later, restaging imaging demonstrated oncological progression, and palliative care was adopted.

This case illustrates the diagnostic and therapeutic challenges of IrP after CRT and durvalumab, particularly when fibrosis predominates, and steroid-refractory progression occurs. Early recognition, close surveillance and timely multidisciplinary management should be warranted, and antifibrotic therapy may be considered in cases of fibrotic evolution despite immunosuppressive treatment.

## Introduction

Durvalumab following concurrent chemoradiotherapy (CRT) is the standard of care for unresectable stage III non-small cell lung cancer (NSCLC) [1,2], which represents approximately one-third of patients with NSCLC at diagnosis [2]. Although durvalumab substantially improves survival outcomes, immune-related pneumonitis (IrP) remains a clinically significant immune-related adverse event (irAE) that can limit the therapeutic benefit of consolidation therapy (i.e., maintenance immunotherapy given after completion of CRT in patients without progression) [2-7]. Differentiating IrP from radiation pneumonitis (RP) is often challenging in the post-CRT setting, as the two entities frequently share overlapping clinical and radiological features. However, they differ in distribution patterns, expected response to immunosuppression, and long-term outcomes [8-10]. While most cases of IrP present with an inflammatory phenotype that is usually steroid-responsive, a small subset evolves into progressive fibrotic lung disease, an uncommon course for which guidance is limited [3,11]. 

This case illustrates a rare and severe course of IrP following CRT and durvalumab consolidation, progressing towards fibrotic lung disease despite timely discontinuation of immunotherapy and administration of high-dose corticosteroids, requiring antifibrotic therapy. It demonstrates the significant diagnostic and therapeutic challenges posed by pneumonitis in the post-CRT setting, particularly when fibrosis predominates, and steroid-refractory progression occurs.

## Case presentation

A 71-year-old man with an Eastern Cooperative Oncology Group (ECOG) performance status of 1, a former smoker with a 25 pack-year history who had quit 13 years earlier, and a history of hypertension and obstructive sleep apnoea, was diagnosed with left lower-lobe (LLL) squamous cell carcinoma (p63-positive, PD-L1 1-5%), stage IIIc (cT3N3M0, locally advanced unresectable disease with extensive nodal involvement), with bronchopulmonary, subcarinal, and bilateral paratracheal nodal metastases. His baseline pulmonary function tests were within normal limits, with forced vital capacity (FVC) 80% of the lower limit of normal (LLN), forced expiratory volume in 1 second (FEV1) 85% of LLN, and diffusing capacity for carbon monoxide (DLCO) 81% of LLN. He received four cycles of carboplatin plus vinorelbine administered concurrently with thoracic radiotherapy (RT) - irradiated areas of LLL mass and left subcarinal, bronchohilar, and paratracheal lymph node regions, total dose of 60 Gy in 30 fractions, corresponding to a lung V20 (percentage of lung volume receiving ≥20 Gy) of 30%. Chest computed tomography (CT) performed 20 days after CRT showed no disease progression or interstitial lung disease, so he was considered eligible for durvalumab consolidation (1500 mg every four weeks), initiated approximately one and a half months after CRT. 

**Figure 1:**
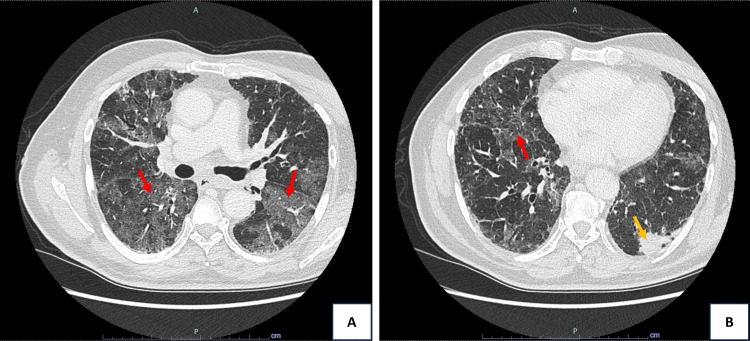
Axial chest CT scans obtained after the second cycle of durvalumab (three months after radiotherapy and two months after initiation of immune checkpoint inhibitor therapy) (A) and (B) show multifocal ground-glass opacities involving all lobes (bilateral patchy inflammatory lung infiltrates (red arrows)), consistent with pneumonitis. A neoformative lesion in the posterobasal segment of the left lower lobe shows no evidence of progression (yellow arrow).

Three weeks after the second durvalumab cycle, the patient developed subacute fatigue and dyspnoea, which progressed over the following two weeks to dyspnoea on minimal exertion, without fever, cough, or sputum. Laboratory analysis showed an elevated C-reactive protein of 77 mg/L, with negative procalcitonin and no leucocytosis. Chest CT revealed multifocal ground-glass opacities (GGO) across all lobes, radiologically consistent with IrP, with no evidence of tumour progression (Figure 1). Bronchoscopy showed no endobronchial lesions, and bronchoalveolar lavage demonstrated marked lymphocytosis (56%) with a normal CD4/CD8 ratio. Microbiological and cytological studies were negative for infection and malignancy, respectively. 

**Figure 2:**
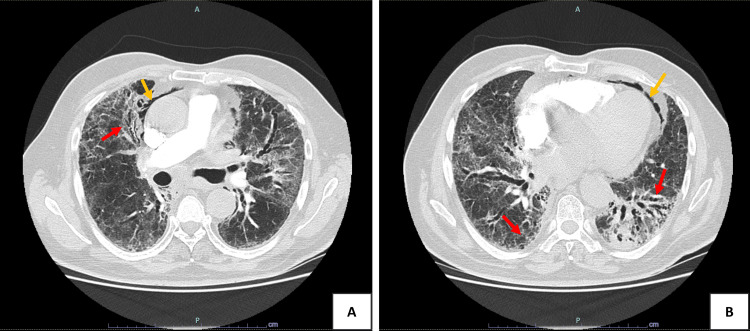
Axial chest CT scans obtained at hospital admission (two months after immune checkpoint inhibitor discontinuation and five months after radiotherapy) (A) and (B) show progression of interstitial involvement, with increased areas of fibrosis and architectural distortion. There is a slight reduction of previous ground-glass opacity, replaced in the same regions by traction bronchiectasis (airway dilatation caused by fibrotic lung remodelling) and parenchymal reticulation of fibrotic character (red arrows). A mild pneumomediastinum is also observed (yellow arrows).

Durvalumab was discontinued, and prednisolone 0.75 mg/kg/day (60 mg/day) was initiated. After a slight initial improvement, the patient experienced clinical deterioration during corticosteroid tapering eight weeks later, at prednisolone doses below 20 mg/day. He developed dyspnea on minimal exertion and new hypoxemic respiratory failure, requiring hospitalisation. Chest CT excluded pulmonary embolism but showed progression of the interstitial involvement with fibrotic evolution and pneumomediastinum (Figure 2).

**Figure 3:**
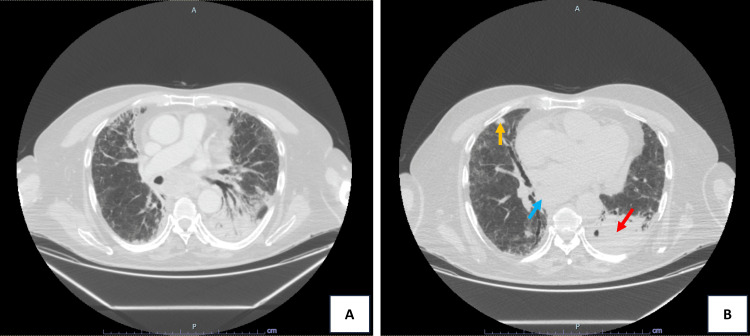
Axial chest CT scans performed for oncologic re-staging (seven months after immune checkpoint inhibitor discontinuation and 10 months after radiotherapy), demonstrating oncological disease progression. (A) and (B) show enlarging mediastinal nodal disease (blue arrow), growth of the primary tumor in the left lower lobe (red arrow) with collapse of the underlying parenchyma, and new multiple bilateral subpleural and pulmonary nodules compatible with metastases (yellow arrow). Motion artifacts limited the assessment of the lung parenchyma; however, there was no evidence of fibrosis progression.

Although fibrotic changes were already predominant, given concern for potentially reversible ongoing inflammatory activity in the setting of clinical worsening during corticosteroid tapering, a multidisciplinary decision was made to administer three daily pulses of 1 g intravenous (IV) methylprednisolone, followed by prednisolone 0.75 mg/Kg. The risks and benefits were carefully weighed, including the risks of infection and aggravation of pneumomediastinum, and cotrimoxazole was started for *Pneumocystis jirovecii* prophylaxis. Despite resolution of respiratory failure, allowing discharge, dyspnoea improved only minimally. Nintedanib was initiated (150 mg twice daily) due to progressive fibrosis despite prior immunomodulatory treatment, but symptoms persisted with significant dyspnea and poor clinical response. A thoracic CT four months later (Figure 3) revealed stability of fibrosis, but oncological progression with enlarging nodal disease, primary tumour growth, and new bilateral pulmonary and pleural metastases. A palliative care approach focused on symptom management was adopted, and nintedanib was suspended.

A detailed timeline of the patient’s clinical course, treatments, and imaging findings is summarised in Table 1.

**Table 1:** Timeline of key clinical events CT: computed tomography; IV: intravenous; CRT: chemoradiotherapy; IrP: immune-related pneumonitis; ICI: immune checkpoint inhibitor

Day 0	Day 42	Day 70	Day 90	Day 101	Day 108	Day 162	Day 169	Day 191	Day 198	Day 313
CRT completion	Durvanumab cycle 1	Durvanumab cycle 2	Onset of dyspnoea	Chest CT with grade 2 IrP (Figure 1)	Prednisolone started at 60 mg/day; ICI discontinuation; slight initial improvement.	Prednisolone tapering to 10 mg/day. Worsening dyspnoea.	Hospitalization for respiratory failure. Chest CT with fibrotic progression (Figure 2) – grade 3 IrP. IV methylprednisolone pulses.	Discharge on prednisolone 50mg/day.	Nintedanib initiated.	Follow-up chest CT with stable fibrosis but oncological progression (Figure 3)

## Discussion

Durvalumab following concurrent CRT has become the standard of care for patients with unresectable stage III NSCLC, as demonstrated in the PACIFIC trial [1,2]. Durvalumab is an immune checkpoint inhibitor (ICI) anti-PD-L1 monoclonal antibody that enhances antitumor immune activity [2]. Despite its survival benefit and generally manageable safety profile, it can cause irAE, among which IrP is the most frequent cause of treatment discontinuation and potentially life‑threatening [2-4]. In the PACIFIC trial, IrP occurred in 9.4% of patients receiving durvalumab, with only 2.7% experiencing grade ≥3 events [2,3,5]. Real‑world studies reported higher incidences and severity (8%-11% grade ≥3), likely reflecting older and frailer populations than those enrolled in clinical trials [6,7].

Risk factors for IrP include older age, poor performance status, smoking history, pre-existing lung disease, impaired pulmonary function, and prior thoracic RT [8-11]. Bilateral or multifocal CT involvement is associated with greater severity [9]. Our patient exhibited several of these factors, like older age, smoking history, prior RT, and bilateral involvement, placing him at increased risk. IrP typically develops within weeks to months after ICI initiation, though late presentations have been reported, even after treatment completion [4,5,10]. The prognostic impact of IrP varies across studies [12,13]. While most patients improve with corticosteroids and ICI discontinuation [4], grade ≥3 IrP has been associated with worse outcomes, including reduced survival [12,13], often due to infections, tumour progression after ICI interruption, or steroid-refractory IrP requiring additional immunosuppression [4].

IrP and RP may coexist, as both RT and ICI recruit and activate lymphocytes, promoting lung inflammation [10]. Differentiating them after CRT is challenging because their clinical and radiological features often overlap, but this distinction is clinically relevant as it influences treatment strategy and steroid intensity, with IrP typically requiring higher-dose and longer immunosuppression and permanent discontinuation of ICI [9]. Both can exhibit cryptogenic organising pneumonia or acute/subacute interstitial pneumonia patterns, while other patterns are less common [14]. However, RP typically shows unilateral changes confined to the radiation field with sharper borders, whereas IrP more often displays bilateral or multifocal involvement with less well-defined borders [9]. These patterns may evolve from an acute inflammatory phase into chronic proliferative organising and fibrotic stages [11].

Only a minority of IrP cases present or progress to a fibrotic phenotype, reported in 0.2% to 7% of cases in some studies [3,9]. The risk factors for this evolution are not well-established, as this phenotype is rare and remains poorly documented. However, available reports suggest that pre-existing lung disease, severe lung involvement, early radiological signs of fibrosis, and persistent inflammation despite immunosuppressive therapy may be associated with progression to fibrosis [11]. Fibrosis may advance despite suppression of inflammation, and current guidelines mainly focus on immunomodulation rather than on managing the chronic fibrosis, leaving treatment largely unsupported by standard protocols [11]. Nintedanib, a multi-targeted tyrosine kinase inhibitor with antifibrotic and potential antitumour activity, has shown potential benefit in selected cases of steroid-refractory or fibrotic IrP [11].

Our patient developed grade 3 IrP, requiring inpatient care and causing major functional impairment. Although the respective contribution of RT and ICI cannot be determined with certainty, the absence of pulmonary infiltrates before durvalumab and the bilateral pattern suggested that IrP was the predominant driver. Emerging serum biomarkers such as Krebs von den Lungen-6 (KL-6) and surfactant protein-D (SP-D) have shown diagnostic and monitoring potential in patients with ICI pneumonitis, but they are not yet widely available in routine clinical practice [15]. This case represents an uncommon progressive course despite durvalumab discontinuation and corticosteroid therapy, leading to a mainly fibrotic pattern on CT rather than persistent inflammatory changes. This radiological evolution supported the use of nintedanib as an antifibrotic agent instead of further immunosuppression, and follow-up imaging demonstrated fibrosis stability, although clinical benefit was limited, and oncological progression occurred. 

## Conclusions

Treating older patients with multimodal therapy requires balancing curative intent against treatment-related toxicity. This case illustrates a rare and severe course of IrP following CRT and durvalumab consolidation, progressing to fibrotic lung disease despite early immunosuppression and ICI discontinuation. Early recognition of pneumonitis through prompt evaluation of new respiratory symptoms with chest CT, bronchoscopy, and exclusion of infection and tumour progression, together with close surveillance, timely multidisciplinary management involving oncology, pulmonology, radiology, and infectious diseases, and awareness of potential fibrotic trajectories, are essential to optimise outcomes and guide treatment in patients with IrP. Further studies are needed to better define the role of antifibrotic therapy in this setting.
